# Supplementary material for: Direct ionic stress sensing and mitigation by the transcription factor NFAT5
Source: bioRxiv. 2023 Sep 24:2023.09.23.559074. Preprint. [Version 1] doi: 10.1101/2023.09.23.559074 (PMC10602047; doi:10.1101/2023.09.23.559074)
Supplement: 1 — Supplementary Figure 1. Optimization and validation of genetic screens for NFAT5 regulators, Related to Figure 1. (A) NFAT5 target gene expression measured by RT-qPCR in wild-type (WT) IMCD3 cells and four independent Nfat5−/−clonal cell lines after 8 hrs in isotonic media (300 mOsm/L) or in hypertonic media (+200 mOsm/L NaCl, raising total media osmolarity to 500 mOsm/L). Black horizontal lines denote mean values calculated from 3 independent measurements shown as points. (B) Abundance of Akr1b3 , Slc5a3, NFAT5 , and the GFP reporter mRNA was measured using RT-qPCR in IMCD3-G cells after exposure to hypertonic stress (+200 mOsm/L NaCl) for the indicated time periods. Each point shows the mean ± Standard Deviation (SD) of three independent measurements. (C) Expression of Akr1b3 and Slc5a3 in WT IMCD3 cells treated with increasing amounts of NaCl added to isotonic media for 8 hrs. Total media osmolarity at each concentration of NaCl is shown in red on the secondary x-axis. Each point shows the mean ± SD of three independent measurements. (D) 8TonE-GFP reporter activity in IMCD3-G cells measured by flow cytometry after 8 hrs in isotonic or hypertonic (+200 mOsm/L NaCl) media in the presence of increasing concentrations of the p38 inhibitors doramapimod or SB 202190. Each point shows the mean ± SD of three independent median measurements, each from a population of >2000 cells. (E) 8TonE-GFP reporter activity measured by flow cytometry in WT IMCD3-G cells or three independent Akap13−/− IMCD3-G clonal cell lines measured after 8 hrs in isotonic or hypertonic (+200 mOsm/L NaCl) media. Each point shows a median measurement from a population of >2000 cells; black horizontal lines show the mean of these independent median values. (F) 8TonE-GFP reporter activity after exposure to hypertonic media (+200 mOsm/L NaCl, 8 hrs) in IMCD3-G cells expressing Cas9 and a non-targeting control sgRNA or sgRNAs targeting the indicated genes on the x-axis. Each point shows an independent me [file NIHPP2023.09.23.559074V1-supplement-1.pdf]

bioRxiv preprint doi: https://doi.org/10.1101/2023.09.23.559074; this version posted September 24, 2023. The copyright holder for this preprint (which was not certified by peer review) is the author/funder, who has granted bioRxiv a license to display the preprint in perpetuity. It is made available under aCC-BY 4.0 International license.

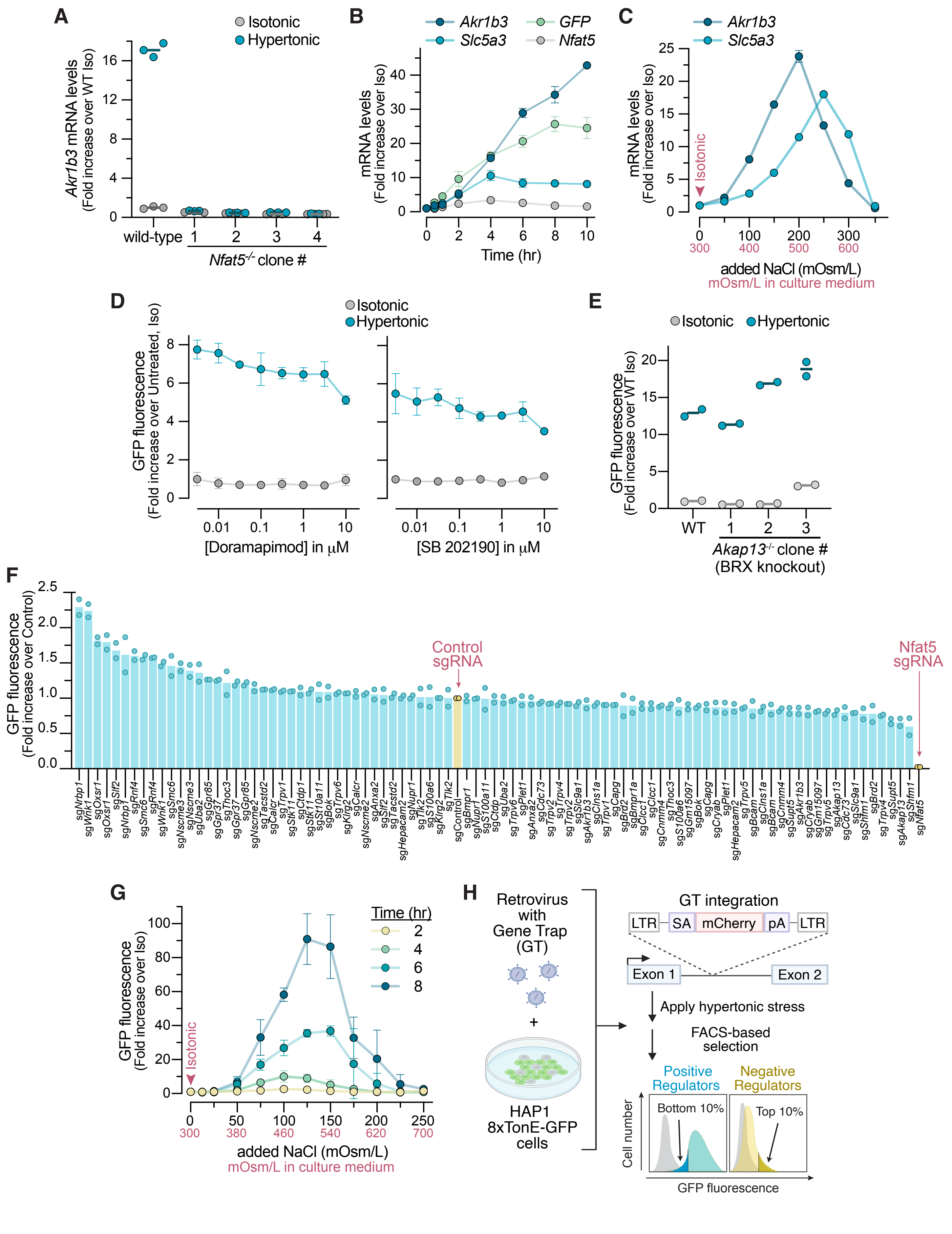

Supplementary Figure 1

bioRxiv preprint doi: https://doi.org/10.1101/2023.09.23.559074; this version posted September 24, 2023. The copyright holder for this preprint (which was not certified by peer review) is the author/funder, who has granted bioRxiv a license to display the preprint in perpetuity. It is made available under aCC-BY 4.0 International license.

Supplementary Figure 1. Optimization and validation of genetic screens for NFAT5 regulators, Related to Figure 1.

(A) NFAT5 target gene expression measured by RT-qPCR in wild-type (WT) IMCD3 cells and four independent *Nfat5*$^{-/-}$ clonal cell lines after 8 hrs in isotonic media (300 mOsm/L) or in hypertonic media (+200 mOsm/L NaCl, raising total media osmolarity to 500 mOsm/L). Black horizontal lines denote mean values calculated from 3 independent measurements shown as points.

(B) Abundance of *Akr1b3*, *Slc5a3*, *Nfat5,* and the *GFP* reporter mRNA was measured using RT-qPCR in IMCD3-G cells after exposure to hypertonic stress (+200 mOsm/L NaCl) for the indicated time periods. Each point shows the mean ± Standard Deviation (SD) of three independent measurements.

(C) Expression of *Akr1b3* and *Slc5a3* in WT IMCD3 cells treated with increasing amounts of NaCl added to isotonic media for 8 hrs. Total media osmolarity at each concentration of NaCl is shown in red on the secondary x-axis. Each point shows the mean ± SD of three independent measurements.

(D) 8TonE-GFP reporter activity in IMCD3-G cells measured by flow cytometry after 8 hrs in isotonic or hypertonic (+200 mOsm/L NaCl) media in the presence of increasing concentrations of the p38 inhibitors doramapimod or SB 202190. Each point shows the mean ± SD of three independent median measurements, each from a population of >2000 cells.

(E) 8TonE-GFP reporter activity measured by flow cytometry in WT IMCD3-G cells or three independent *Akap13*$^{-/-}$ IMCD3-G clonal cell lines measured after 8 hrs in isotonic or hypertonic (+200 mOsm/L NaCl) media. Each point shows a median measurement from a population of >2000 cells; black horizontal lines show the mean of these independent median values.

(F) 8TonE-GFP reporter activity after exposure to hypertonic media (+200 mOsm/L NaCl, 8 hrs) in IMCD3-G cells expressing Cas9 and a non-targeting control sgRNA or sgRNAs targeting the indicated genes on the x-axis. Each point shows an independent median measurement from a population of >2000 cells.

(G) 8TonE-GFP reporter activity in human haploid (HAP1) cells at the indicated time points in response to increasing amounts of NaCl added to isotonic media. Each point shows the mean ± SD of three independent median measurements, each from a population of >2000 cells. Total media osmolarity at each concentration of NaCl is shown in red on the secondary x-axis.

(H) Strategy used for the retroviral insertional mutagenesis screen in human HAP1 cells using the stably integrated 8TonE-GFP reporter. Insertion of the gene-trap (GT) cassette, positioned between the retroviral long terminal repeats (LTRs), in the sense orientation into an intron will trigger transcriptional termination: the splice acceptor (SA) will cause this cassette to be spliced to the preceding exon, leading to transcriptional termination due to a strong polyadenylation (pA) signal. Fluorescence activated cell sorting (FACS) was used to collect cells carrying mutations in genes encoding positive or negative regulators of the NFAT5 transcriptional response following exposure to hypertonic stress (see **Figure 1F**).

bioRxiv preprint doi: https://doi.org/10.1101/2023.09.23.559074; this version posted September 24, 2023. The copyright holder for this preprint (which was not certified by peer review) is the author/funder, who has granted bioRxiv a license to display the preprint in perpetuity. It is made available under aCC-BY 4.0 International license.

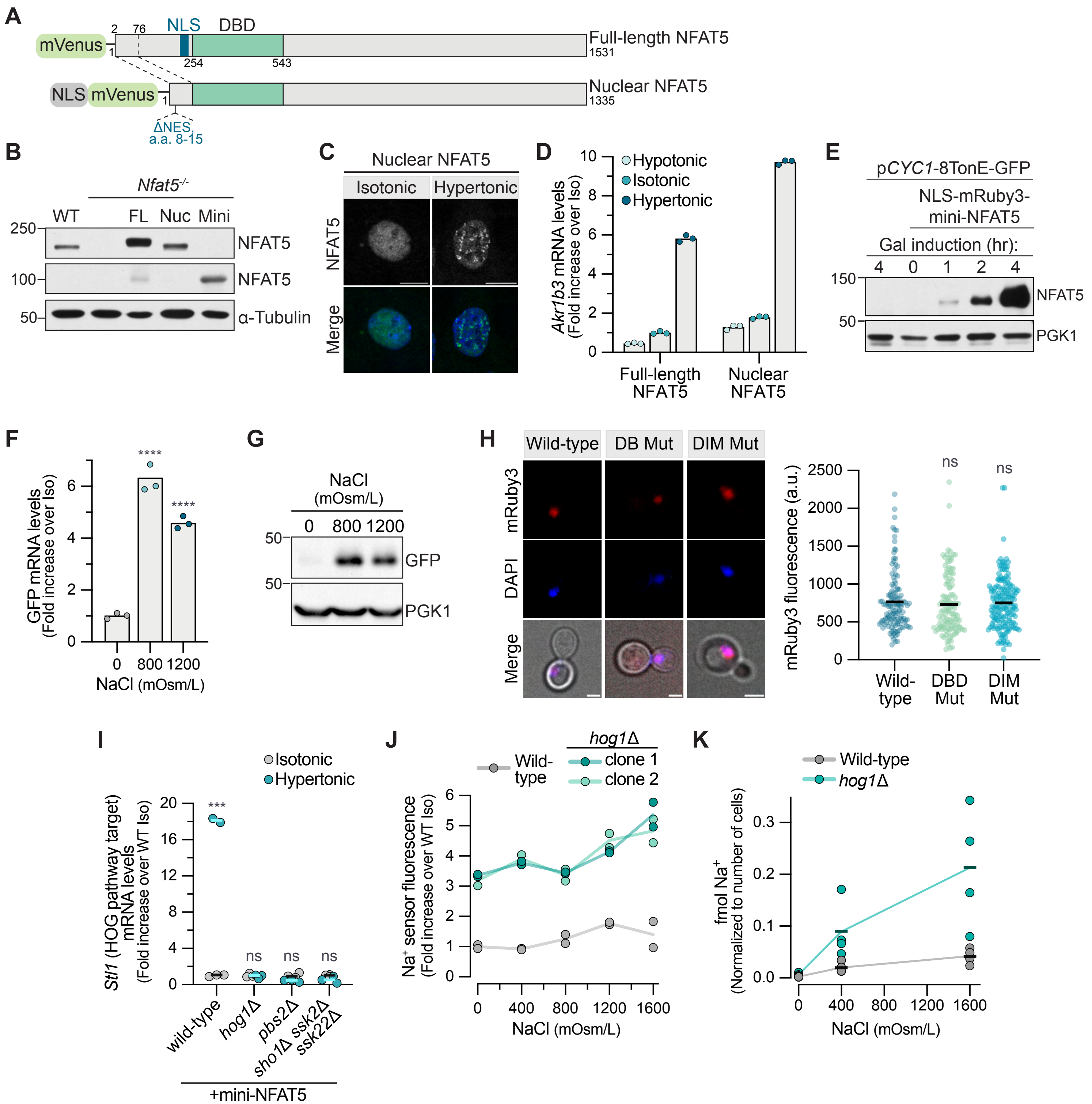

Supplementary Figure 2

bioRxiv preprint doi: https://doi.org/10.1101/2023.09.23.559074; this version posted September 24, 2023. The copyright holder for this preprint (which was not certified by peer review) is the author/funder, who has granted bioRxiv a license to display the preprint in perpetuity. It is made available under aCC-BY 4.0 International license.

Supplementary Figure 2. Characterization of mini-NFAT5 in IMCD3 and yeast cells, Related to Figure 2.

(A) Diagram of full-length, mVenus-tagged NFAT5 (top) compared to a variant (Nuclear NFAT5) constitutively targeted to the nucleus by removal of its endogenous nuclear import and export sequences (a.a. 77-253) and addition of a heterologous strong nuclear localization sequence (NLS).

(B) Immunoblot comparing NFAT5 abundance in WT, *Nfat5*$^{-/-}$, or *Nfat5*$^{-/-}$ IMCD3 cells stably expressing full-length (FL), nuclear (nuc), or mini-NFAT5.

(C) Subcellular localization of nuclear NFAT5 after 30 min in isotonic or hypertonic (+200 mOsm/L NaCl) media. Fluorescence signal from the mVenus tag fused to NFAT5 is shown alone (top) and merged with a DNA stain (DAPI) to mark nuclei (bottom). Scale bar: 10 µm.

(D) Expression of an NFAT5 target gene in *Nfat5*$^{-/-}$ cells stably expressing full-length or nuclear NFAT5 after 8 hrs in hypotonic, isotonic, or hypertonic media (+200 mOsm/L NaCl).

(E) Abundance of NLS-mRuby3-mini-NFAT5 at various times after galactose addition in a yeast strain used to test activation of NFAT5 by hypertonic stress. This strain contains two stably integrated transgenes, one encoding NLS-mRuby3-mini-NFAT5 driven by a galactose-inducible promoter and a second encoding the 8TonE-*pCYC1*-GFP NFAT5 reporter (see **Figure 2D**). The leftmost lane shows a control strain lacking the mini-NFAT5 transgene but containing the reporter.

(F) Abundance of GFP mRNA in yeast cells expressing the 8TonE-*pCYC1*-GFP reporter and NLS-mRuby3-mini-NFAT5 was measured by RT-qPCR after 2 hrs in CSM containing the indicated concentrations of NaCl.

(G) Abundance of GFP protein in yeast cells expressing the 8TonE-*pCYC1*-GFP reporter and NLS-mRuby3-mini-NFAT5 was measured by immunoblotting after 4 hrs in CSM containing the indicated concentrations of NaCl.

(H) Representative images (left) showing subcellular localization of wild-type NLS-mRuby3-mini-NFAT5 and variants carrying mutations that abrogate DNA binding (DB Mut) or dimerization (DIM Mut) (see **Figure 2G**) in yeast cells used in the experiment depicted in **Figure 2H**. Graph on right shows quantitation of nuclear mRuby3 fluorescence in single yeast cells (measured from images of the type shown on the left) expressing the indicated NFAT5 variant (n>118 cells per strain).

(I) Expression of the HOG pathway target gene *Stl1* in wild-type, *hog1*Δ, *pbs2*Δ, or *ssk*2Δ *ssk22*Δ *sho1*Δ cells after 2 hrs in CSM supplemented with 1200 mOsm/L NaCl.

(J) Fluorescence of a sodium sensor (see methods) in wild-type and *hog1*Δ cells (2 independent clones) in response to increasing amounts of NaCl added to CSM. Two technical replicates were measured for each clone and are shown separately.

(K) Abundance of sodium ions was measured by inductively coupled plasma optical emission spectroscopy (ICP-OES) in wild-type and *hog1*Δ cells.

Statistics: Bars (**D**,**F**) or horizontal lines (**H**,**I,K**) denote mean values calculated from independent measurements shown as points. Statistical significance was determined by a one-way (**F**) or two-way (**I**) ANOVA test with Sidak's multiple comparison post-test (n>3 independent experiments) or (**H**) by the Kruskal Wallis test. p-values symbols are: **** <0.0001 and *** <0.001.

bioRxiv preprint doi: https://doi.org/10.1101/2023.09.23.559074; this version posted September 24, 2023. The copyright holder for this preprint (which was not certified by peer review) is the author/funder, who has granted bioRxiv a license to display the preprint in perpetuity. It is made available under aCC-BY 4.0 International license.

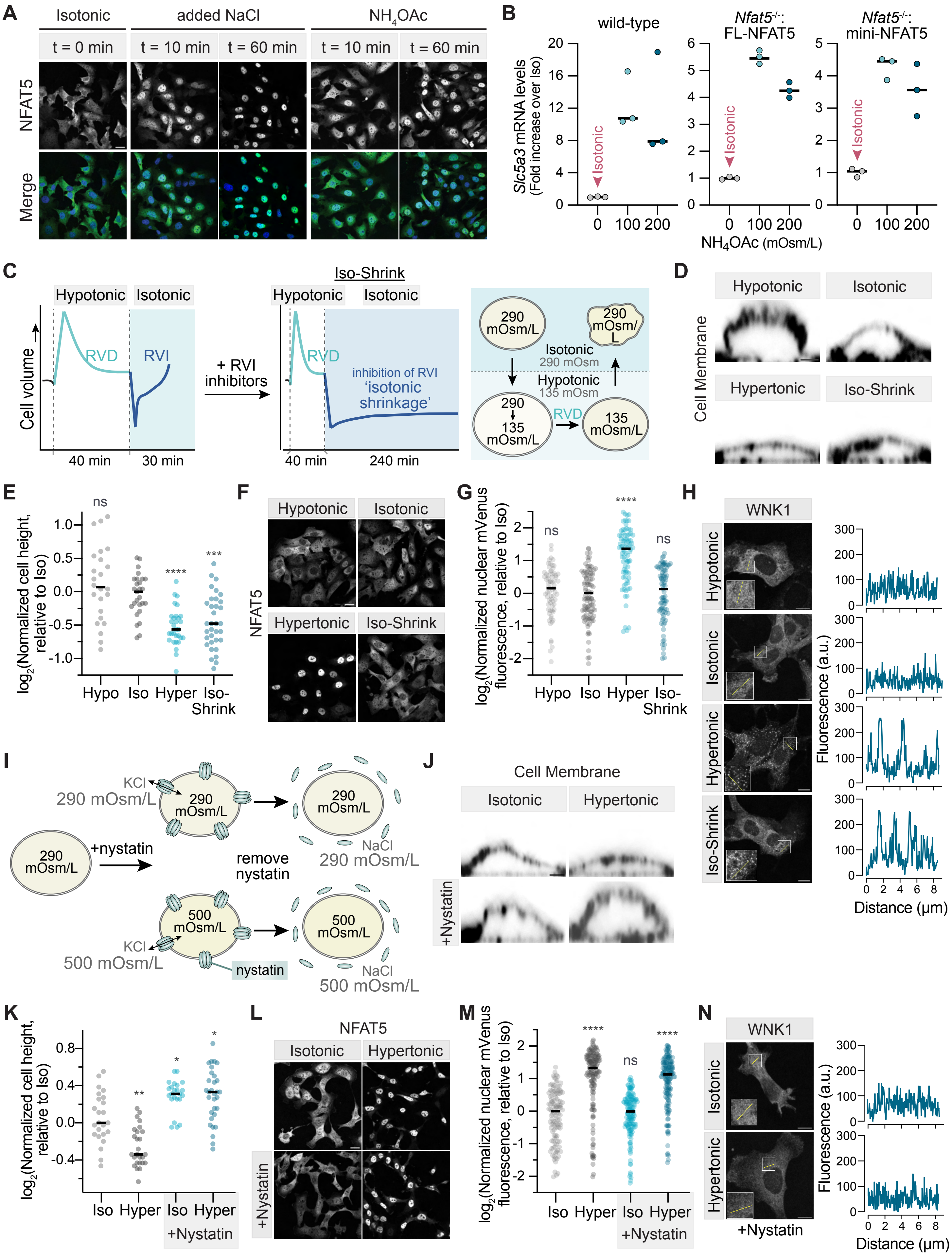

Supplementary Figure 3

bioRxiv preprint doi: https://doi.org/10.1101/2023.09.23.559074; this version posted September 24, 2023. The copyright holder for this preprint (which was not certified by peer review) is the author/funder, who has granted bioRxiv a license to display the preprint in perpetuity. It is made available under aCC-BY 4.0 International license.

Supplementary Figure 3. The effects of cell shrinkage and ionic stress on NFAT5 and WNK1, Related to Figure 3.

(A) The subcellular localization of stably expressed mVenus-NFAT5 (top) after 10 or 60 min in hypertonic media (+200 mOsm/L NaCl or NH_4OAc). Fluorescence signal from the mVenus tag fused to NFAT5 is shown alone (top) or merged with a nuclear stain (DAPI, bottom). Images like these were used for the quantitative analysis of NFAT5 nuclear accumulation shown in **Figure 3I**. Scale bar: 20 µm.

(B) Expression of an NFAT5 target gene in WT IMCD3 cells or *Nfat5*$^{-/-}$ IMCD3 cells stably expressing mVenus-tagged full-length or mini-NFAT5 6 hrs after the addition of NH_4OAc to isotonic media (see **Figure 3J** for a similar measurement using the 8TonE-GFP NFAT5 reporter). Black horizontal lines mark the median of three independent measurements, each shown as a point.

(C) Diagram depicting the principle behind the isotonic shrinkage (Iso-Shrink) protocol, which allows cell shrinkage under isotonic conditions. Cells subjected to a hypotonic shock initially swell, but then restore their volume through regulatory volume decrease (RVD), which is caused by loss of osmotically active cytoplasmic ions and small molecules. The subsequent transfer of cells to isotonic media causes cell shrinkage since the cytoplasmic osmolarity is lower than media osmolarity. The addition of regulatory volume increase (RVI) inhibitors prevents cell volume recovery following isotonic shrinkage. Schematic taken from reference [45].

(D) Confocal images of IMCD3 cells in the xz plane stained with CellMask to highlight the plasma membrane. Cells were exposed to hypotonic (~200 mOsm/L), isotonic (~300 mOsm/L), and hypertonic (~500 mOsm/L with the addition of 200 mOsm/L NaCl) conditions, or subjected to the isotonic shrinkage protocol (bottom right Iso-Shrink panel) shown in **Figure S3C** and described in the methods. Scale bar: 2 µm.

(E) The height of IMCD3 cells (*n*>24 cells per condition, measured from images of the type shown in **Figure S3D**) after the isotonic shrinkage protocol (**Figure S3C)** compared to cells in hypotonic, isotonic, or hypertonic media.

(F) Subcellular localization of mVenus-NFAT5 stably expressed in IMCD3 cells following isotonic shrinkage, compared to cells in hypotonic, isotonic, or hypertonic media. Scale bar: 20 µm.

(G) Nuclear mVenus fluorescence was measured (using images of the type shown in **Figure S3F**) from single IMCD3 cells (*n*>69 cells per condition) stably expressing full-length mVenus-NFAT5 following isotonic shrinkage, compared to cells in hypotonic, isotonic, or hypertonic media.

(H) Distribution of GFP fluorescence in *Wnk1*$^{-/-}$ IMCD3 cells stably expressing GFP-WNK1 after isotonic shrinkage, compared to its distribution in cells exposed to hypotonic, isotonic, or hypertonic media. Line scans show fluorescence intensity traces along the trajectories of the lines shown in the insets. Scale bar: 10 µm.

(I) The ionophore nystatin forms ion conducting pores in the plasma membrane that allow equilibration of intracellular and extracellular osmolarity. When nystatin-treated cells are exposed to hypertonic media, intracellular ion concentrations rise without collateral cell shrinkage and macromolecular crowding.

(J) Confocal images of IMCD3 cells in the xz plane stained with CellMask to highlight the plasma membrane. Cells were exposed to isotonic or hypertonic media in the presence or absence of nystatin. Scale bar: 2 µm.

(K) The height of IMCD3 cells (*n*>23 cells per condition, measured from images of the type shown in **Figure S3I**) after exposure to isotonic or hypertonic media in the presence or absence of nystatin.

(L) Subcellular localization of mVenus-NFAT5 in IMCD3 cells after exposure to isotonic or hypertonic media in the presence or absence of nystatin. Scale bar: 20 µm.

(M) Nuclear mVenus fluorescence was measured (using images of the type shown in **Figure S3L**) from single IMCD3 cells (*n*>121 cells per condition) stably expressing full-length mVenus-NFAT5 after exposure to isotonic or hypertonic media in the presence or absence of nystatin.

(N) Subcellular distribution of GFP-WNK1 following nystatin treatment in isotonic or hypertonic media. Line scans correspond to fluorescence intensity traces along the trajectories of the lines in the inset. Scale bar: 10 µm.

bioRxiv preprint doi: https://doi.org/10.1101/2023.09.23.559074; this version posted September 24, 2023. The copyright holder for this preprint (which was not certified by peer review) is the author/funder, who has granted bioRxiv a license to display the preprint in perpetuity. It is made available under aCC-BY 4.0 International license.

Statistics: Circles in (**E**,**G**,**K**,**M**) denote measurements from single cells and the black horizontal lines mark the median of the populations. Statistical significance (**E**,**G**,**K**,**M**) of differences in comparison to the isotonic condition was determined by a Kruskal-Wallis test with Dunn's multiple comparison test (*n*>3 independent experiments). P-value symbols are: **** *p*-value<0.0001, *** *p*-value<0.001, ** *p*-value<0.01, and * *p*-value<0.05.

bioRxiv preprint doi: https://doi.org/10.1101/2023.09.23.559074; this version posted September 24, 2023. The copyright holder for this preprint (which was not certified by peer review) is the author/funder, who has granted bioRxiv a license to display the preprint in perpetuity. It is made available under aCC-BY 4.0 International license.

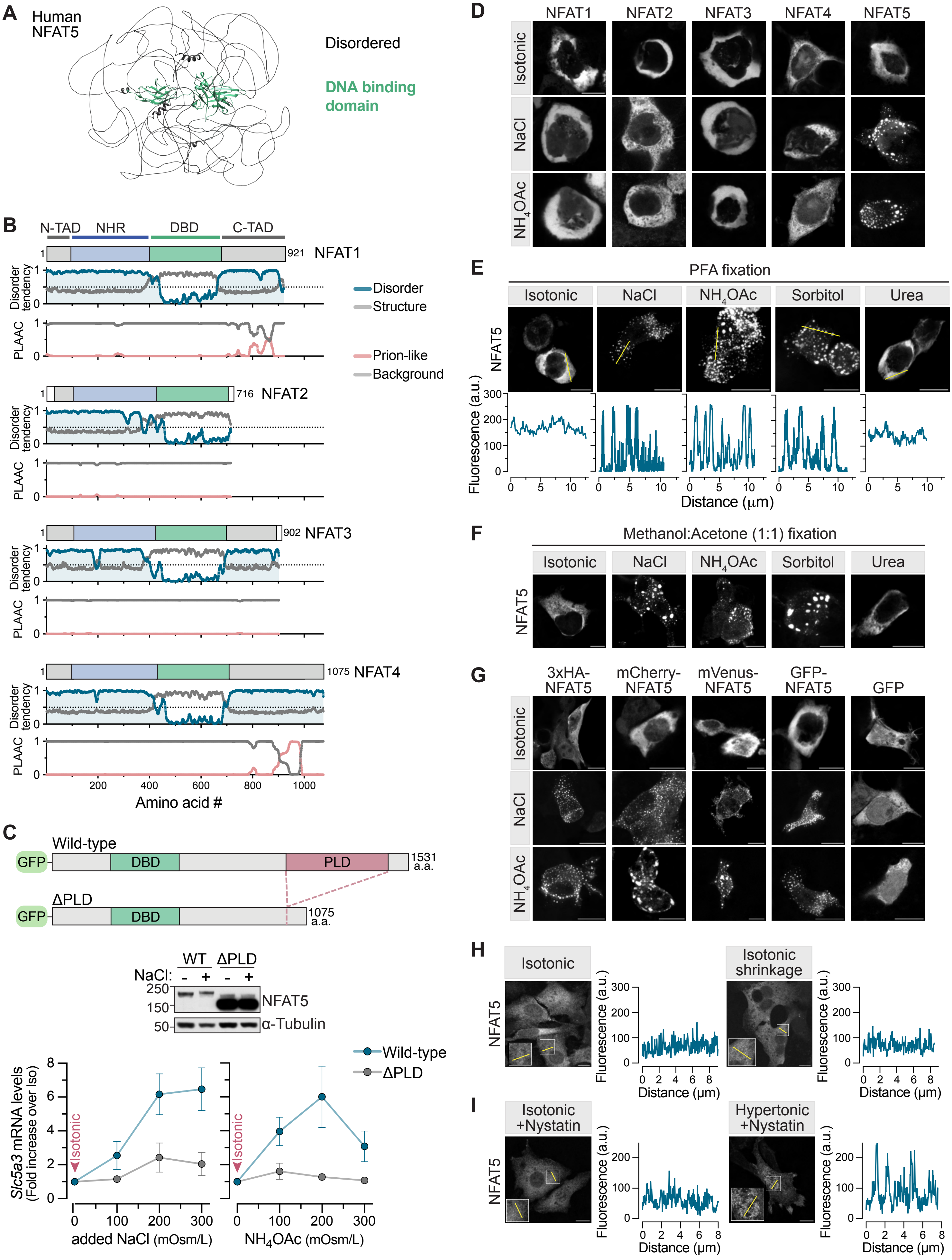

Supplementary Figure 4

bioRxiv preprint doi: https://doi.org/10.1101/2023.09.23.559074; this version posted September 24, 2023. The copyright holder for this preprint (which was not certified by peer review) is the author/funder, who has granted bioRxiv a license to display the preprint in perpetuity. It is made available under aCC-BY 4.0 International license.

Supplementary Figure 4. Formation of NFAT5 droplets in response to stress, Related to Figure 4.

(**A**) AlphaFold prediction of the mostly disordered human NFAT5 (Uniprot ID: O94916) structure.[94]

(**B**) Domain structures, disorder tendency and predicted prion-like domains (PLDs) in human NFATs1-4 (compare to NFAT5 shown in **Figure 4A**). NFATs1-4 share a conserved NFAT-homology region (NHR) and DNA binding domain (DBD, also conserved with NFAT5) flanked by N- and C-terminal transactivation domains (TAD).

(**C**) Expression of the NFAT5 target gene *Slc5a3* (bottom graphs) in *Nfat5*$^{-/-}$ IMCD3 cells stably expressing wild-type GFP-NFAT5 (WT) or a variant lacking the PLD (GFP-NFAT5 ΔPLD) 6 hrs after the addition of increasing amounts of NaCl (left) or NH_4OAc (right) to isotonic media. Each point represents the mean ± SD of three independent measurements. Cartoon (top) shows the domain structure of the NFAT5 variants and immunoblot (middle) shows the abundances of each protein.

(**D**) Distribution of GFP-tagged NFATs1-5 transiently transfected into 293T cells 30 min after the addition of NaCl or NH_4OAc (+100 mOsm/L each) to isotonic media.

(**E,F**) Distribution of GFP-NFAT5 in transiently-transfected 293T cells 30 min after the addition of NaCl, NH_4OAc, sorbitol or urea (+100 mOsm/L each) to isotonic media. Cells were fixed with paraformaldehyde (PFA) in (**E**) or Methanol/Acetone in (**F**). Line scans in (**E**) correspond to fluorescence intensity traces along the trajectories of the yellow lines on the images.

(**G**) Distribution of NFAT5 fused to different epitope tags in 293T cells after the addition of NaCl or NH_4OAc (+100 mOsm/L each, 30 min) to isotonic media. Localization of GFP alone (without fusion to NFAT5) is shown in the rightmost column.

(**H,I**) Distribution of GFP-NFAT5 stably expressed in *Nfat5*$^{-/-}$ IMCD3 after isotonic shrinkage (**H**) or nystatin treatment (**I**) in the presence of an isotonic or hypertonic solution (see **Figures S3C** and **S3I** and Methods for details). Line scans correspond to fluorescence intensity traces along the trajectories of the lines shown in the insets.

Scale bars for panels (**D-I**): 10 μm.

bioRxiv preprint doi: https://doi.org/10.1101/2023.09.23.559074; this version posted September 24, 2023. The copyright holder for this preprint (which was not certified by peer review) is the author/funder, who has granted bioRxiv a license to display the preprint in perpetuity. It is made available under aCC-BY 4.0 International license.

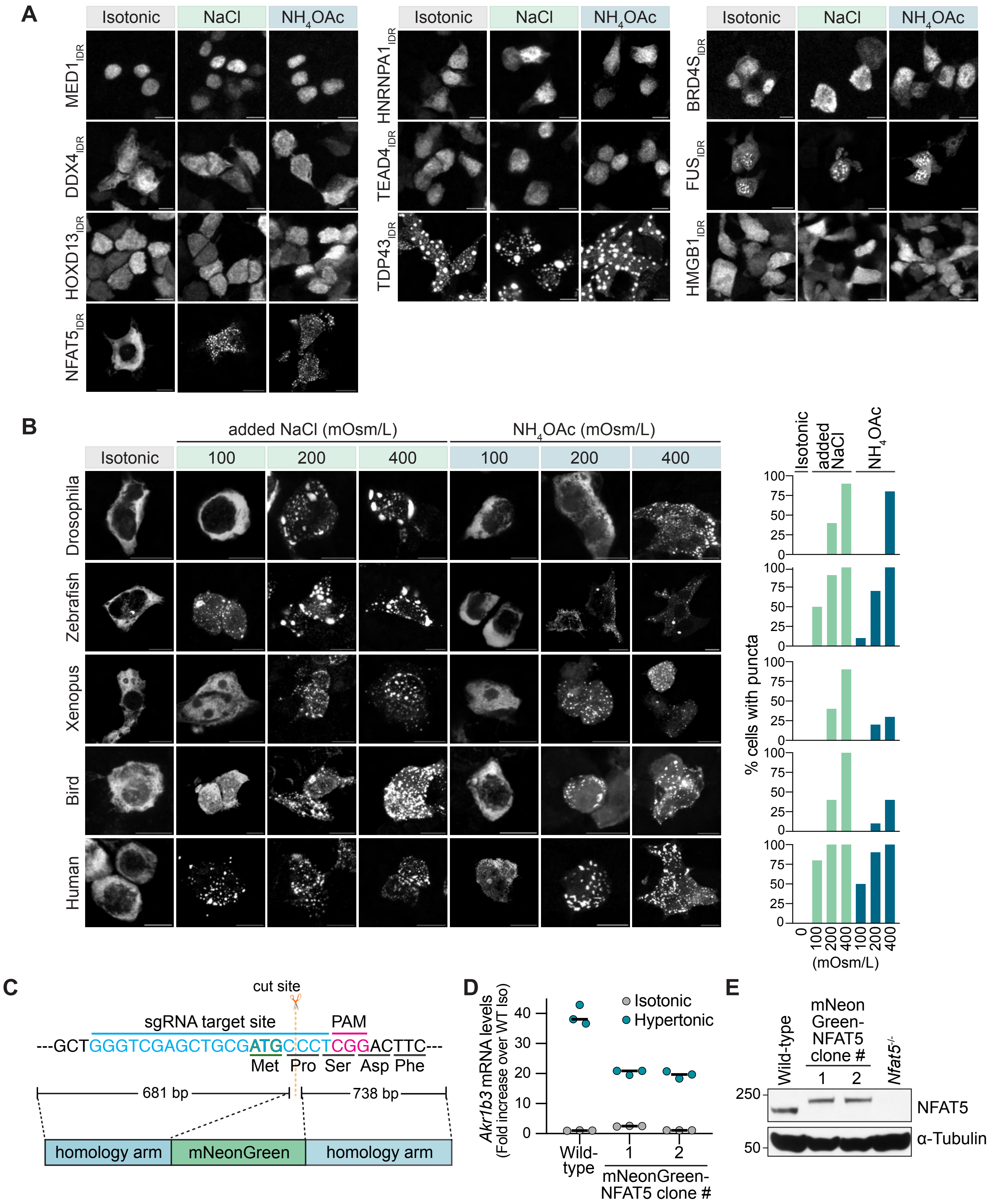

Supplementary Figure 5

bioRxiv preprint doi: https://doi.org/10.1101/2023.09.23.559074; this version posted September 24, 2023. The copyright holder for this preprint (which was not certified by peer review) is the author/funder, who has granted bioRxiv a license to display the preprint in perpetuity. It is made available under aCC-BY 4.0 International license.

Supplementary Figure 5. Evolutionarily conserved ionic stress sensing by NFAT5, Related to Figure 4.
(**A**) Distribution of the GFP-tagged NFAT5 C-terminal IDR (CTD, **Figure 2A**) compared to Fluorescent Protein-tagged IDRs from nine unrelated proteins after transient transfection into 293T cells. Cells were fixed and imaged 30 min after the addition of NaCl or NH_4OAc (+100 mOsm/L each) to isotonic media.
(**B**) Distribution of chimeric NFAT5 proteins transiently transfected into 293T cells. Chimeras were generated by replacing the human NFAT5 CTD (a.a. 544-1531) with corresponding CTDs from insect (*Drosophila melanogaster*, a.a. 542-1210), fish (*Danio rerio*, a.a. 534-1257), amphibian (*Xenopus laevis*, a.a. 489-1408) and bird (*Columba livia*, a.a. 496-1455) NFAT5 homologs. Cells were imaged 30 min after addition of the indicated concentrations of NaCl or NH_4OAc to isotonic media. Bar graphs on the right show the percentage of transfected cells with puncta at each salt concentration (*n*>210 cells evaluated per condition).
(**C**) Exon 1 sequence of the mouse *Nfat5* gene targeted by the sgRNA used to insert the mNeonGreen coding sequence in IMCD3 cells using homology-directed repair. The Protospacer Adjacent Motif (PAM) and site of predicted cleavage by Cas9 are shown above the sequence; the donor template for homologous recombination is shown below.
(**D**) Expression of the NFAT5 target gene *Akr1b3* in wild-type IMCD3 cells or two clonal *mNG-Nfat5* knock-in cell lines after 8 hrs in isotonic or hypertonic media (+200 mOsm/L NaCl). Black horizontal lines denote the median from three independent measurements shown as points.
(**E**) Insertion of the mNG tag at the N-terminus of both *Nfat5* alleles in IMCD3 cells was confirmed by immunoblotting.
Scale bars for panels (**A**,**B**): 10 µm.

bioRxiv preprint doi: https://doi.org/10.1101/2023.09.23.559074; this version posted September 24, 2023. The copyright holder for this preprint (which was not certified by peer review) is the author/funder, who has granted bioRxiv a license to display the preprint in perpetuity. It is made available under aCC-BY 4.0 International license.

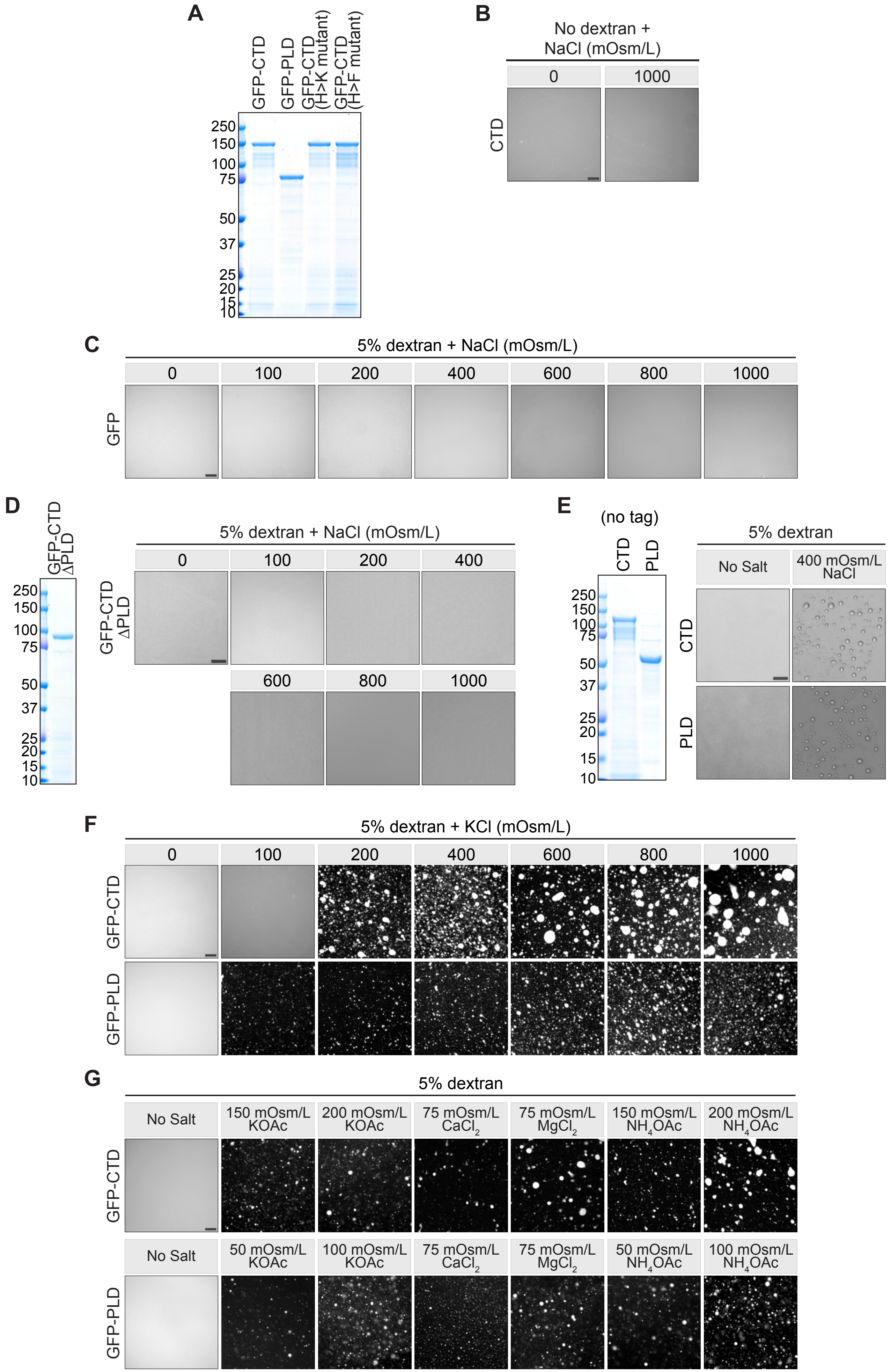

Supplementary Figure 6

bioRxiv preprint doi: https://doi.org/10.1101/2023.09.23.559074; this version posted September 24, 2023. The copyright holder for this preprint (which was not certified by peer review) is the author/funder, who has granted bioRxiv a license to display the preprint in perpetuity. It is made available under aCC-BY 4.0 International license.

Supplementary Figure 6. Characterization of the *in vitro* droplet formation assay of NFAT5 protein fragments, Related to Figure 5.

(A,D,E) Coomassie-stained polyacrylamide gels showing the purity of proteins used for *in vitro* droplet formation assays in **Figures 5**, **6** and **7**. Molecular weight standards in kilodaltons (kDa) are indicated to the left.

(**B**) Fluorescence microscopy was used to assess droplet formation *in vitro* by purified GFP-CTD (70 μM) in buffered solution without Dextran.

(**C,D**) Fluorescence microscopy was used to assess droplet formation *in vitro* by 100 μM purified GFP or GFP-CTD-ΔPLD (CTD lacking the PLD, see **Figure S4C**) at the indicated concentrations of NaCl.

(**E**) Brightfield microscopy was used to assess droplet formation by untagged NFAT5 CTD (70 μM) and PLD (90 μM).

(**F,G**) *In vitro* droplet formation by GFP-NFAT5 CTD (70 μM, top row) or GFP-NFAT5 PLD (90 μM, bottom row) at increasing concentrations of KCl (**F**) or in the presence of a variety of different salts (**G**). All solutions contained 5% dextran.

Scale bars for panels (**B-G**): 5 μm.

bioRxiv preprint doi: https://doi.org/10.1101/2023.09.23.559074; this version posted September 24, 2023. The copyright holder for this preprint (which was not certified by peer review) is the author/funder, who has granted bioRxiv a license to display the preprint in perpetuity. It is made available under aCC-BY 4.0 International license.

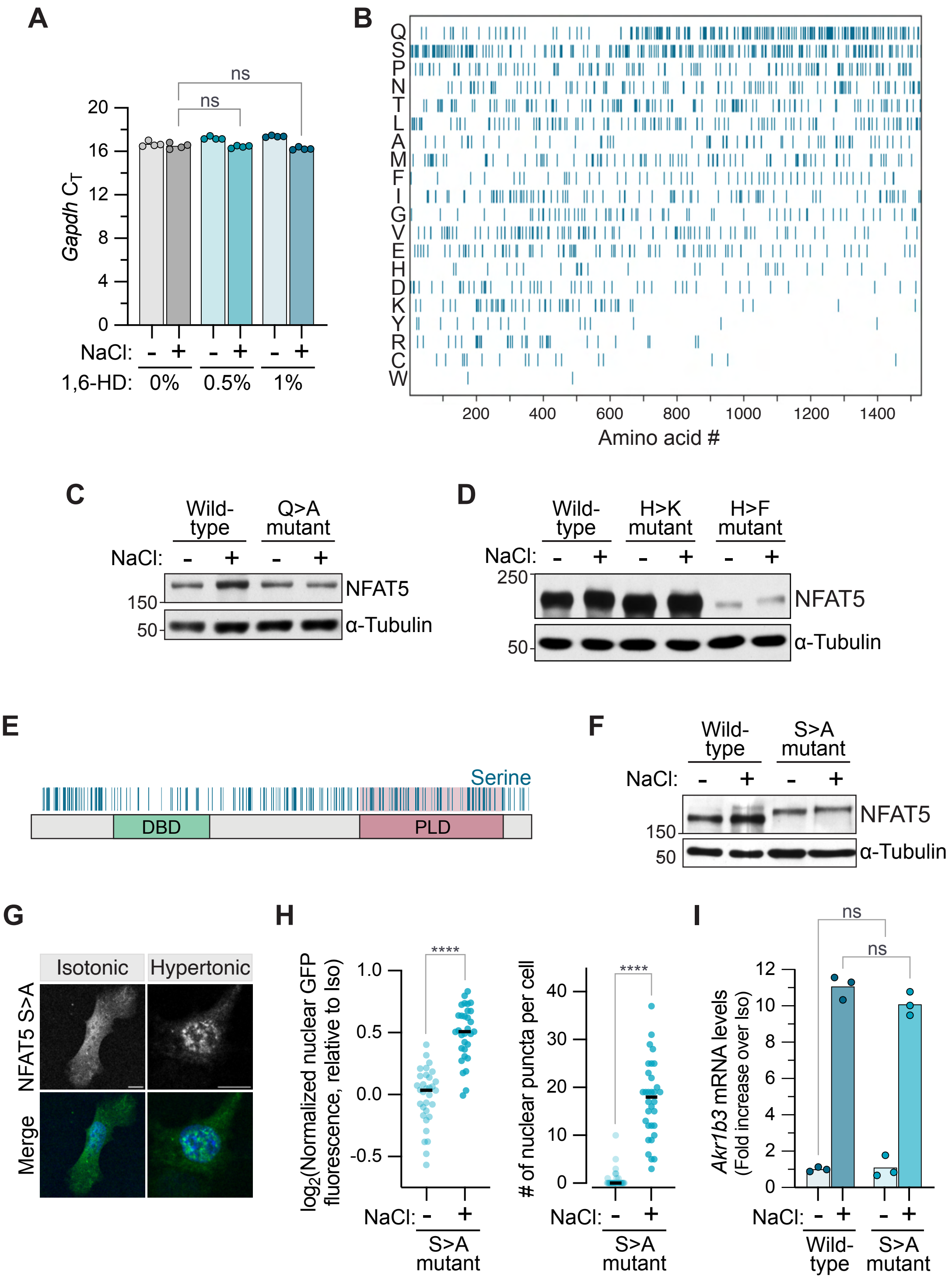

Supplementary Figure 7

bioRxiv preprint doi: https://doi.org/10.1101/2023.09.23.559074; this version posted September 24, 2023. The copyright holder for this preprint (which was not certified by peer review) is the author/funder, who has granted bioRxiv a license to display the preprint in perpetuity. It is made available under aCC-BY 4.0 International license.

Supplementary Figure 7. Related to Figure 6.
(A) Abundance of *Gapdh* mRNA (measured by its C_T value in a RT-qPCR assays) after 6 hrs of exposure to 0, 0.5, or 1% 1,6-HD in isotonic or hypertonic (+200 mOsm/L NaCl) media. Compare to the effect of 1,6-HD on *Akr1b3* mRNA abundance in **Figure 6B**. Bars show the mean of 3 measurements.
(B) Position of each of the 20 a.a. in the NFAT5 linear sequence (oriented along the x-axis). In each row, every occurrence of a single amino acid is marked by a vertical blue line.
(C, D) Immunoblot comparing the abundances of WT GFP-NFAT5 to the GFP-NFAT5_QA, GFP-NFAT5_HK and GFP-NFAT5_HF mutants.
(E) Positions of all serine residues in NFAT5 are marked by vertical blue lines. All serine residues in the PLD (highlighted in red) were mutated to alanine residues in the GFP-NFAT5_SA protein.
(F) Immunoblot comparing abundances of WT GFP-NFAT5 to the GFP-NFAT5_SA mutant.
(G) Subcellular distribution of GFP-NFAT5_SA stably expressed in $Nfat5^{-/-}$ IMCD3 cells after 30 min in hypertonic media (+200 mOsm/L NaCl). Fluorescence signal from NFAT5 is shown alone (top) and merged with DAPI to mark nuclei (bottom). Images of the type shown in (**G**) were used in (**H**) to measure nuclear NFAT5 fluorescence (left) and number of NFAT5 nuclear puncta (right) per cell (n>30 cells, with black horizontal line showing the median and each point showing the measurement in one cell).
(I) Expression of the NFAT5 target gene *Akr1b3* in IMCD3 cells stably expressing WT GFP-NFAT5 or the GFP-NFAT5_SA mutant after 8 hrs in isotonic or hypertonic (+200 mOsm/L NaCl) media. Bars denote the mean of 3 measurements, each shown as points.
Scale bars for panel (**G**): 10 µm
Statistics: For (**A**,**I**), statistical significance was determined by a two-way ANOVA test, Sidak's multiple comparison. For (**H**) statistical significance was determined by a Kruskal-Wallis test, Dunn's multiple comparison. P-value symbols are: **** p-value<0.0001.
